# The Role of Attachment in Gambling Behaviors and Gambling Disorder: A Systematic Review

**DOI:** 10.1007/s10899-022-10163-1

**Published:** 2022-11-02

**Authors:** Simon Ghinassi, Silvia Casale

**Affiliations:** 1 Department of Experimental and Clinical Medicine, University of Florence, Largo Brambilla, 3, 50134 Florence, Italy; 2 Department of Health Sciences, Psychology Unit, University of Florence, Via di San Salvi, 12, 50135 Florence, Italy

**Keywords:** Addictive behaviors, Attachment, Attachment-related phenomena, Behavioral addictions, Gambling, Systematic review

## Abstract

In recent years, a growing number of attachment-based studies have contributed to the understanding of both substance and behavioral addictions. Although gambling is a form of addictive behavior widespread all over the world, both among young people and adults, the evidence on the association between attachment-related phenomena and gambling has not yet been systematized in literature. The aim of the present study, therefore, is to provide a systematic literature review aimed at summarizing the empirical evidence on this topic. Following the updated 2020 PRISMA guidelines, a systematic search in four electronic scientific databases (Scopus, PubMed, PsycInfo and Web of Science) was conducted. After removing duplicates, 146 records were double-screened, with 12 articles meeting the inclusion criteria. Additionally, by means of a backward search a further article was selected. Altogether, 13 articles were selected for the present systematic review. With few exceptions, the results underline the significant role played by attachment-related phenomena in gambling behaviors, highlighting that specific attachment contexts have a different influence on gambling, also depending on whether gamblers are youths or adults. In particular, while secure attachment has proven to be a protective factor for the onset of gambling behavior, insecure attachment has emerged to be a vulnerability factor in two ways. On the one hand, it directly favors gambling behaviors; on the other, it affects coping strategies and the individual’s ability to identify and regulate emotions, which in turn predict gambling. Limitations, strengths, and implications of the present systematic review are discussed.

## Introduction

### Gambling Behaviors and Gambling Disorder

Gambling has existed since the dawn of human civilization and is currently a very popular activity around the world, so much so that it is even considered a socially acceptable form of entertainment in many countries (Calado & Griffiths, 2016; Molinaro et al., 2018). Since gambling is considered a typical adult activity, it is not surprising that most adults have gambled at some point in their lives (Calado & Griffiths, 2016). However, recent literature highlights the popularity and spread of gambling even among adolescents, which is alarming since legislation generally prohibits minors from participating in any form of gambling (Andrie et al., 2019; Calado et al., 2017a; Emond & Griffiths, 2020). The growing diffusion of gambling among both adults and adolescents is probably due to the extraordinary technological developments that have characterized the last decades and that have allowed the proliferation of a great variety of easily available and accessible gambling activities (Derevensky & Gilbeau, 2019).

Although many adolescents and adults engage in gambling in a non-regular or recreational manner (Black & Shaw, 2019; Emond et al., 2020; Tani et al., 2021), some individuals develop cognitive and behavioral symptoms (e.g., preoccupation with gambling, difficulties in quitting gambling, financial problems, and relationship breakdowns). The persistent and recurrent pattern of gambling associated with substantial distress or impairment is referred to as Pathological Gambling in the Diagnostic and Statistical Manual of Mental Disorder-IV-Text Revised (DSM-IV-TR) and is included in the Impulse-control disorders not elsewhere classified section (American Psychiatric Association, 2000). However, even though pathological gambling shares some characteristic features with the other disorders in this section (trichotillomania, intermittent explosive disorder, kleptomania, and pyromania), such as for example not resisting impulses or temptations to perform a harmful act and pleasure or a sense of release while performing the act, there is little empirical evidence on the association between this disorder and the others included in this section (Petry et al., 2014). Conversely, there is much evidence that underlines the similarities between gambling and addictive disorders, not only in diagnostic, clinical and neurological terms, but also in their treatment and comorbidity (Petry et al., 2014). For these reasons, this disorder was moved into the category of Substance-Related and Addictive Disorders changing its name to Gambling Disorder in the DSM-5 (APA, 2013). To date, Gambling Disorder is the only recognized behavioral addiction in the DSM-5.

Gambling disorder is characterized by the presence of persistent and recurrent problem gambling behaviors that involve a wide range of negative consequences from a personal, familiar, social, financial, legal, study and employment point of view (APA, 2013). However, since there is ample evidence that there are many individuals who suffer gambling-related harm but do not meet diagnostic criteria for gambling disorder, other terms are also widely used in literature, such as at-risk and problem gambling. These terms designate a subclinical condition typically less serious than the gambling disorder, but which also has negative consequences for the individual's well-being (Hodgins et al., 2011; Hunt & Blaszczynski, 2019).

In view of the diffusion of gambling worldwide (e.g., Çakıcı et al., 2021) and the plethora of negative consequences of such behavior (see for a discussion Langham et al., 2015), literature has focused on exploring the possible individual, relational and community risk and protective factors related to the onset, maintenance and severity of gambling behavior, notwithstanding the presence of differences in pre-existent psychopathology, maladaptive personality traits, and motives to gamble among gamblers. Alcohol, tobacco and cannabis use, personality traits (i.e., impulsivity and sensation-seeking), depression, male gender, and gambling-related variables (i.e., number of gambling activities and problem gambling severity) have been shown to be related to problem gambling (for a review, see Dowling et al., 2017). When it comes to adolescents, problem gambling is more likely to occur among males, ethnic minority, and those who have parents who gambled and did not live with both parents (for a review, see Calado et al., 2017a).

In recent years, a growing number of attachment-based studies have tried to contribute to the understanding of gambling (or, at least, specific subtypes of gambling, see Blaszczynski & Nower, 2002), in keeping with emerging evidence suggesting a developmental pathway leading from attachment to addictive behaviors, including problematic patterns of substance use (Schindler, 2019; Schindler & Bröning, 2015) and compulsive Internet-related behaviors (for systematic reviews see D'Arienzo et al., 2019; Musetti et al., 2022).

### Attachment-Related Phenomena

In his pioneering work, Bowlby (1969, 1973, 1980) has argued that early experiences of caregiving influence the individual's adaptation and maladaptation "from the cradle to the grave." According to attachment theory, human beings are endowed with an innate attachment behavioral system that motivates and regulates the behavior of the child that use proximity-seeking as a primary inborn strategy for regulating affect. In other words, the child seeks the physical and psychological proximity of the attachment figure, believed to be able to offer care, with the intent of seeking safety. This system, which performs a protective function from threats and relieves stress, is not always active, but remains silent in all those safe situations in which the child perceives the surrounding environment as safe and the attachment figure as present and responsive. When the attachment figure is available and reactive in the moment of need, there is an optimal functioning of the attachment system and the structuring of a secure attachment in the child. Conversely, if the attachment figure does not respond to the child’s needs, proving insensitive, unreliable, or inconsistent, and failing to provide adequate relief from distress, this promotes a sense of attachment insecurity. In both cases the child develops Internal Working Models (IWMs), of the self and others, as a consequence of the internalization of repeated relational experiences with the attachment figure (Bowlby, 1969). IWMs are mental representations that have the function of conveying the perception and interpretation of events by the individual, allowing one to make predictions and create expectations about the events of one's relational life (Bowlby, 1969; Main et al., 1985). The first empirical contribution to Bowlby's attachment theory was made by Ainsworth et al. (1978), who developed the Strange Situation, a "20-min miniature drama" (Bretherton, 1992) aimed at measuring and classifying the child's attachment. Based on the information gathered by means of this procedure, three major categories of attachment styles were described: secure, insecure avoidant, and insecure ambivalent/resistant. Subsequently, Main and Solomon (1986) added a fourth category of attachment style, called disorganized-insecure attachment. A secure attached child, who experiences an attachment figure who is responsive to their own needs (i.e., a secure base), will have a perception of self as worthy of love and care, and of others as reliable and available. Conversely, an insecure attached child, with an insensitive, unreliable, or inconsistent attachment figure, will develop insecure but organized IWMs of self as unacceptable, unlovable, and unworthy, and of others as hurtful, rejecting, or unsafe. Specifically, insecure avoidant children are independent of the attachment figure, both physically and emotionally, and do not seek contact when distressed (Behrens et al., 2007). The attachment figure of these children turns out to be rejecting and emotionally detached, systematically pushing their children away in response to their requests for closeness and contact (Main & Stadtman, 1981). On the other hand, ambivalent/resistant insecure children adopt an ambivalent behavioral style towards the attachment figure. These children commonly exhibit clingy and dependent behavior but reject the attachment figure when engaging in interaction by crying inconsolably in some circumstances (Cassidy & Berlin, 1994). Insecure ambivalent children fail to develop any feeling of security from the attachment figure as the latter responds unpredictably to the child's requests and is therefore potentially unreliable in times of difficulty (Pederson & Moran, 1996). Finally, children with a disorganized attachment style appear to be disoriented, stunned, or confused. This attachment style is often observed in abused children and results in an inability to integrate IWMs into a coherent structure (Benoit, 2004). Abusive attachment figures expose their children to a pervasive paradox as they are, at the same time, the only source of comfort and relief from distress and the main source of fear (Van Ijzendoorn et al., 1999).

Over time, attachment theory has been extended from infant-mother relationships to adult relationships, and numerous categorical and dimensional conceptualizations have been proposed (Ravitz et al., 2010). However, some authors have highlighted that the different attachment conceptualizations found in previous studies were attributable to two higher-order dimensions, namely attachment anxiety and attachment avoidance (Bartholomew, 1990; Bartholomew & Horowitz, 1991). Attachment anxiety is characterized by excessive sensitivity to perceived threat to self and relationships, fear of abandonment and rejection, and excessive desire for approval from others, while attachment avoidance is characterized by the rejection of intimacy and discomfort due to proximity, as well as the difficulty in trusting others by relying only on oneself (Mikulincer et al., 2004). Thus, secure attachment is characterized by low levels of these two dimensions, dismissing attachment by high levels of avoidance and low levels of anxiety, preoccupied attachment by high anxiety and low avoidance and, finally, fearful attachment by high levels in both dimensions.

Furthermore, attachment can be considered in the light of two different theoretical models: trait and context-specific model of attachment (Caron et al., 2012). The trait model conceptualizes attachment as a personality characteristic of an individual and therefore relatively stable throughout the life span (Fraley, 2002; McConnell & Moss, 2011). The context-specific model conceptualizes attachment as a context-specific variable dependent on relationship (with parents, partner, and friends), meaning that individuals may have multiple mental models of their attachment patterns that can vary depending on the significant other they interact with (Caron et al., 2012; Cozzarelli et al., 2000). In the field of behavioral addictions, such a distinction has been very recently taken into account in systematic reviews of the empirical literature concerning attachment and problematic social networking site use (Musetti et al., 2022).

### Attachment and Gambling

Although insecure attachment is not per se a pathological condition, it represents a vulnerability factor to psychopathology (Herstell et al., 2021; Mikulincer & Shaver, 2012, 2019) and its relationship to addictions is widely supported by the empirical literature. Insecure attachment affects the ability to regulate emotions (Fuchshuber et al., 2019; Pascuzzo et al., 2015), and addiction is considered as a disorder in self-regulation and as an attempt at self-medication (Casale & Fioravanti, 2017; Khantzian, 2003; Marlatt & Donovan, 2005). In this perspective, addictive behaviors represent a way to replace the “self-object needs” (Kohut, 1971) with a drug, an activity (e.g., gambling) or an object that can help overcome a feeling of emptiness (Flores, 2004). In other words, drug use or repetitive involvement in a rewarding non-substance-related behavior can make up for a lack of intimacy (Estévez et al., 2017; Flores, 2004), giving the unrealistic feeling of having a secure base (Höfler & Kooyman, 1996), to the point that some authors view addictive behaviors as an attachment disorder (Flores, 2004; Gill, 2018; MacMillan & Sisselman-Borgia, 2018). In sum, individuals with insecure attachment, having difficulty regulating their emotions, are more prone to engage in addictive behaviors, such as gambling, as a way to satisfy their attachment needs (Flores, 2004). In fact, there is a huge number of studies which support the hypothesized link between attachment and addictive behaviors (e.g., Burgkart et al., 2022).

In keeping with this perspective, numerous studies have highlighted the role of gambling behaviors as an external regulatory modality of negative emotional states (Di Trani et al., 2017; Rogier & Velotti, 2018; Velotti et al., 2021)—i.e., the motivation of some gamblers for their behaviors is based on an effort to modulate negative affective states. Moreover, both anxious or avoidant attachment have been related to a greater severity of gambling (Keough et al., 2018), and the impact of insecure attachment styles has also been identified in community samples (Calado et al., 2017a, 2017b). Thus, one possible developmental pathway to gambling might involve attachment and related negative feelings (Gori et al., 2021), in accordance with findings showing that some gamblers present with premorbid anxiety and/or depression and negative developmental variables and life events (see Blaszczynski & Nower, 2002).

### The Present Study

To our knowledge there is currently no systematic review of the literature on the association between attachment and gambling. Hence, our aim is to present a systematic literature review that synthesizes the available evidence on this topic. As we want to take into account the different theoretical approaches in the field of attachment, we will use the expression "attachment-related phenomena" as an umbrella term that includes the variety of constructs relating to attachment proposed over time (for an overview see Musetti et al., 2022). Moreover, we will examine this association by considering adolescents/young adults and adults separately. This choice is due to the fact that adolescents and adults differ in terms of the type and importance they give to significant others. In fact, while in adolescence the relationship with parents and peers is of primary importance, in adulthood the most important reference figure appears to be the romantic partner (Fraley & Davis, 1997). Consequently, the population age might make a difference in studies that adopted the context-specific attachment model.

Therefore, we will report the studies conducted so far that have investigated the relationship between attachment and gambling, taking into consideration the theoretical model of attachment considered (trait model versus context-specific model), the population under investigation (adolescents/young adults or adults and general or clinical population), and the possible mediators that may influence this relationship.

## Method

The present systematic review was conducted following the updated guidelines of the Preferred Reporting Items for Systematic Reviews and Meta-Analyses (PRISMA) statement (Page et al., 2021) with no time restrictions. The systematic literature search was done on January 1, 2022, it being updated for the last time on January 31, 2022.

### Eligibility Criteria

Studies had to meet the following inclusion criteria to be included in the systematic literature review: (1) original quantitative research; (2) published in a peer-reviewed journals written in English; (3) explore and report the relationship between attachment and gambling or compare a clinical group of individuals diagnosed with pathological gambling/gambling disorder with a control group; 4) investigate both attachment and gambling through reliable and validated measures. Studies concerning attachment to school and general social context were excluded.

### Information Sources and Search Strategies

Two authors (SC and SG) conducted the systematic literature search. The following databases were searched: Scopus, PubMed, PsycInfo, and Web of Science. The search strategy was narrowed down to Titles, Abstracts, and Keywords, and included the following search string: "attachment" AND (“gambl*” OR “betting” OR “wager*” OR “behavioral addiction”). The reference list of relevant articles and reviews was scrutinized to identify potential additional articles.

### Identification, Selection, and Quality Assessment of Studies

First, two authors (SC and SG) conducted independent research on the databases mentioned above. Subsequently, the included studies were critically appraised using the Appraisal tool for Cross-Sectional Studies (AXIS tool) (Downes et al., 2016). This tool comprises 20 elements with three response options (*Yes* = 1, *No* = 0, *Don’t know* = 0) aimed to evaluate the introduction, methods, results, discussion, and other aspects (e.g., Conflicts of Interest and Ethical Approval) of the study. A subjective quality score is generated from the sum of the scores obtained, ranging from 0 to 20. According to Moor and Anderson (2019), scores ranging from 0 to 7 indicate low quality, scores ranging from 8 to 14 indicate medium quality, and scores ranging from 15 to 20 indicate high quality.

### Data Extraction

Data extraction was done by one author (SG) and checked by another (SC). For each selected study, the following information was extracted: (1) authors and year of publication; (2) country; (3) study design; (4) characteristics of the sample; (5) measures of attachment-related phenomena and gambling; (6) type of conceptualization of attachment (trait or context-specific model); (7) key results. Moreover, the measures used in the studies included in the review, the constructs they assessed, and the frequency of their use were extracted.

## Results

**Fig. 1:**
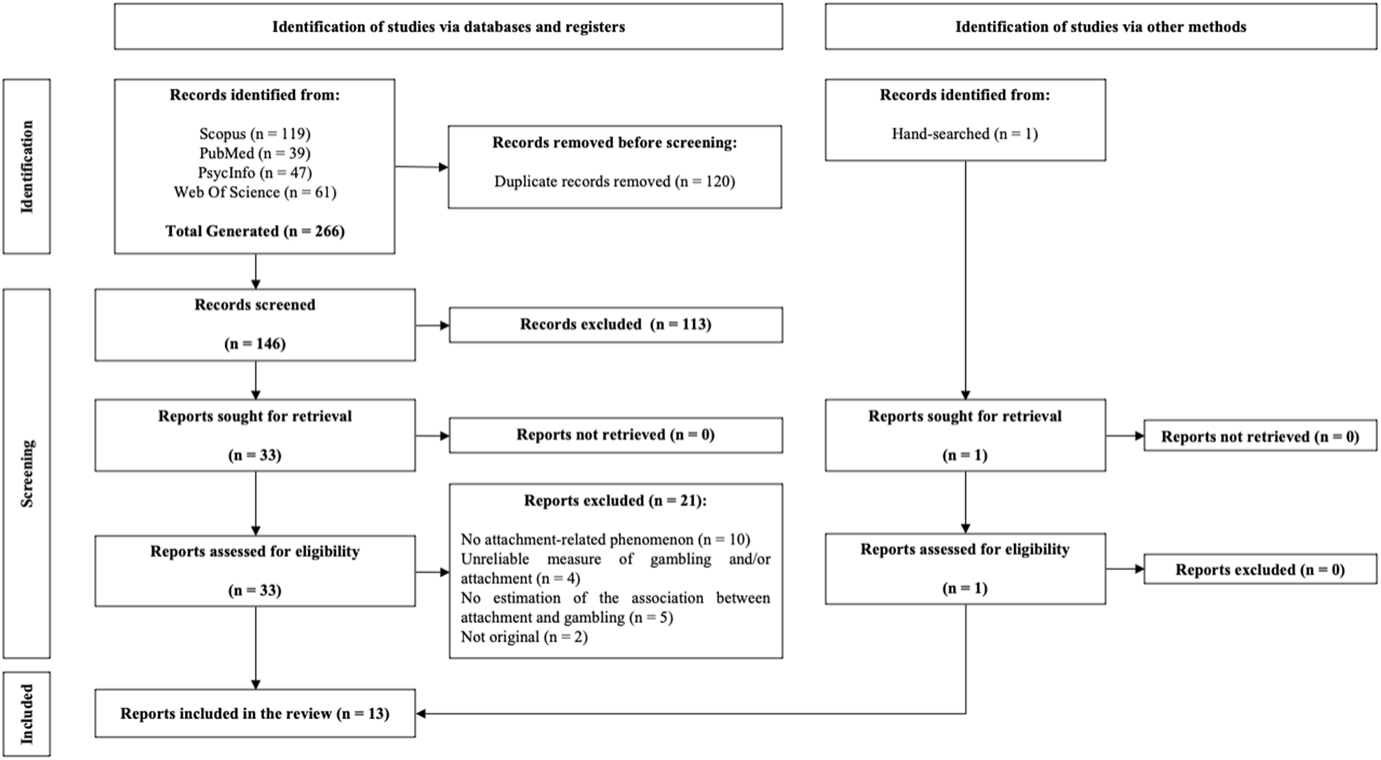
PRISMA flowchart depicting the study selection process

The details of the selection process are illustrated in Fig. 1.

The independent research on the databases led to the identification of 266 records. Subsequently, duplicates (*n* = 120) were removed and the titles and abstracts of the remaining 146 records were double screened. Articles deemed unsuitable by both reviewers (based on title and abstracts) were excluded. After this preliminary screening, 33 full-text records were examined for eligibility assessment. Even in this case, two authors independently conducted the eligibility assessment. This process led to the selection of 12 articles and, by backward search, a further article was selected. Therefore, a total of 13 articles were selected based on the inclusion criteria used for the present systematic review.

### Studies Characteristics

The quality assessment is detailed in Table 1. Scores for individual studies ranged from 14 to 17 (*M* = 15.92, *SD* = 0.73), with 12 studies scoring in the high range and one in the medium range. Table 2 shows the data extracted from the 13 selected studies for the present systematic review. The 13 studies adopted a cross-sectional design and covered 5,061 participants (*M* = 389.31), with sample sizes ranging from 60 to 1,137. Six studies were conducted in Italy, two in Spain, one in England and Portugal simultaneously, and one each in United States, Canada, Turkey, England. Seven studies recruited an adolescent/young adult sample, while six studies focused on adults. Regarding the studies conducted with samples of adults, four studies recruited patients with a diagnosis of pathological gambling or gambling disorder, one recruited patients with a diagnosis of "Substance-Related and Addictive Disorder", and one focused on patients with a lifetime diagnosis of a depressive disorder or a bipolar disorder.

**Table 1:** Quality assessments and total scores using the AXIS tool (Downes et al., 2016)

Authors (years)	Q1	Q2	Q3	Q4	Q5	Q6	Q7	Q8	Q9	Q10	Q11	Q12	Q13*	Q14	Q15	Q16	Q17	Q18	Q19*	Q20	Total quality score/20	Quality rating
Calado et al. (2017b)	Y	Y	N	Y	Y	N	N	Y	Y	Y	Y	Y	N	*na*	Y	Y	Y	Y	N	Y	16	High
Calado et al. (2020)	Y	Y	N	Y	Y	N	N	Y	Y	Y	Y	Y	N	*na*	Y	Y	Y	Y	N	Y	16	High
Caretti et al. (2018)	Y	Y	N	Y	Y	N	N	Y	Y	Y	Y	Y	N	Y	Y	Y	Y	Y	N	Y	17	High
Di Trani et al. (2017)	Y	Y	N	Y	Y	N	N	Y	Y	Y	Y	Y	N	*na*	Y	Y	Y	N	N	Y	15	High
Estévez et al. (2021)	Y	Y	N	Y	Y	N	N	Y	Y	Y	Y	Y	N	*na*	Y	Y	Y	Y	N	Y	16	High
Estévez, et al. (2017)	Y	Y	N	Y	Y	N	N	Y	Y	Y	Y	Y	N	*na*	Y	Y	Y	Y	N	Y	16	High
Kaya and Deveci (2021)	Y	Y	N	Y	Y	N	N	Y	Y	Y	Y	Y	N	*na*	Y	Y	Y	Y	N	Y	16	High
Keough et al. (2018)	Y	Y	N	Y	Y	N	N	Y	Y	Y	Y	Y	N	*na*	Y	Y	Y	Y	N	Y	16	High
Magoon and Ingersoll (2006)	Y	Y	N	Y	Y	N	N	Y	Y	Y	Y	Y	Y	N	Y	Y	Y	Y	*na*	Y	14	Medium
Pace et al. (2013)	Y	Y	Y	Y	Y	Y	N	Y	Y	Y	Y	Y	N	*na*	Y	Y	Y	Y	*na*	Y	17	High
Ponti et al. (2021)	Y	Y	N	Y	Y	N	N	Y	Y	Y	Y	Y	N	*na*	Y	Y	Y	Y	N	Y	16	High
Terrone et al. (2021)	Y	Y	N	Y	Y	N	N	Y	Y	Y	Y	Y	N	*na*	Y	Y	Y	Y	N	Y	16	High
Tonioni et al. (2014)	Y	Y	N	Y	Y	N	N	Y	Y	Y	Y	Y	N	*na*	Y	Y	Y	Y	N	Y	16	High
Mean																					15.92	
Standard Deviation																					0.73	

Y = Yes, N = No, *na* = Don’t know

^*^Reverse coded

**Table 2:** Studies on gambling and attachment included in the review (n = 13)

Authors (years)	Country	Sample	Measures	Attachment theoretical model	Findings	Quality rating/20
Calado et al. (2017b)	England	*n* = 988 adolescents and young adults (males = 59.2%)	*Attachment*: AAQ; *problem gambling*: DSM-IV-MR-J	Contextual (parents)	**Correlation analysis**	16
		Age range = *na* (*M* = 19.8, *SD* = 2.0)			No significant correlations between the three attachment dimensions (Angry distress, Availability, Goal-corrected partnership) and problem gambling were found	
					**Logistic regression analysis**	
					Attachment did not show a significant relationship with problematic gambling	
					**Structural equation model**	
					Emotion-focused coping fully mediated the relationship between angry distress and problem gambling (*B* = 0.13; 95% CI = 0.06, 0.20)	
Calado et al. (2020)	Portugal and England	*n* = 1137 adolescents and young adults: 552 from Portugal [males = 46.4%; Age range = *na* (*M *= 18.2, *SD* = 2.4)]; 585 from England (males = 57.8%; Age range = *na* (*M* = 19.1, S*D *= 1.8)	*Attachment*: AAQ; *Problem gambling*: DSM-IV-MR-J	Contextual (parents)	**Structural equation model**	16
					In both Portuguese and English samples, the attachment dimensions of angry distress, availability, and goal-corrected partnership were indirectly associated with problem gambling through sensation seeking	
					Portuguese sample: angry distress indirect effect (*b* = 0.58, *SE* = 0.24, 95% *BCa CI* = 0.30, 1.59); indirect effect of availability (*b* = − 2.03, *SE* = 0.99, 95% *BCa CI* = − 7.36, − 0.96); indirect effect of goal-corrected partnership (*b* = 1.66, *SE *= 0.81, 95% *BCa CI* = 0.79, 5.49)	
					English sample: angry distress indirect effect (*b* = 0.72, *SE* = 0.37, 95% *BCa CI* = 0.32, 1.97); indirect effect of availability (*b *= − 1.66, *SE* = 0.91, 95% *BCa CI* = − 4.84, −0.72); indirect effect of goal-corrected partnership (*b* = 1.11, *SE* = 0.67, 95% *BCa CI* = 0.44, 3.56)	
Caretti et al. (2018)	Italy	*n* = 698 subjects (males = 69.05%): 515 in clinical sample with diagnosis of “Substance-Related and Addictive Disorder” [males = 77.28%; Age range = *na* (*M* = 39.03, *SD* = 11.64)]; 183 in control sample [males = 45.90%; Age range = *na* (*M* = 29.09, *SD* = 8.62)]	*Attachment: *ABQ – seven domains addiction scale	Trait and Contextual (partner)	**Correlation analysis**	17
			(Domain 1, Separation anxiety) and PTI-ASS; *Gambling*: ABQ – Severity Index and SOGS		Secure, preoccupied, avoidant and unresolved scales of PTI-ASS were not significantly correlated with gambling assessed with Severity Index	
					Attachment assessed with Seven Domains Addiction Scale was not significantly correlated with scores of SOGS	
Di Trani et al. (2017)	Italy	*n* = 60 participants with Gambling Disorder (males = 80%). Age range = 18–68 (*M* = 44.53, *SD* = 13.00)	*Attachment: *ECR-R; *Intensity of Gambling Disorder*: KFG	Contextual (partner)	**Correlation analysis**	15
					Avoidance and anxiety attachment were positively correlated with severity of gambling (*r* = .30, *p* = .02; *r* = .38, *p* < .001, respectively)	
					**Linear multiple regression analysis**	
					Attachment anxiety predicted the severity of gambling (*R*^2^ change = 0.18; *β* = 0.40; *p* = .003)	
					**Analysis of variance**	
					Fearful group scored higher than the secure group (*F* = 2.30; *p* = .001; *d* = 1.08) in gambling severity	
Estévez et al. (2021)	Spain	*n* = 599 adolescents and young adults [(males = 53.6%)	*Attachment to parents and peer*: IPPA; *Gambling disorder*: SOGS-RA	Contextual (mother, father, and peers)	**Student’s *t***	16
		Age range = 12–21 (*M* = 15.35, *SD* = 1.79)]: 547 not gamblers and non-problem gamblers [males = 52.9%; Age range = *na* (*M* = 15.28, *SD* = 1.78)]; 52 problem gamblers [males = 61.5%; Age range = *na* (*M* = 16.12, *SD* = 1.76)]			Problem gamblers reported significantly lower strength of parental attachment as compared to non-problem gamblers (mother; *t*(485) = 2.23, *p *< .05, *d* = − 0.37; father (*t*(482) = 2.47, *p *< .05; *d* = −0.40)	
					No significant differences emerged between problem gambler and non-problem gambler on the level of peer attachment	
					**Correlation analysis**	
					Gambling was negatively associated to mother (*r* = − .10, *p* < .05) and father (*r* = − .09, *p* < .05) attachment, whereas peer attachment was not significantly associated with gambling (*r* = − .05, *p* = *ns*)	
					**Multiple mediation analyses**	
					Difficulties in identifying emotions was found to mediate the link between maternal attachment and gambling	
Estévez et al. (2017)	Spain	*n* = 472 students (males = 48.4%); Age range = 13–21 (*M* = 15.6, *SD* = 1.33)	*Attachment to parents and peer*: IPPA; *Gambling disorder*: SOGS-RA	Contextual (mother, father, and peers)	**Correlation analysis**	16
					Father attachment (but not mother and peer attachment) was negatively correlated with gambling disorder (*r* = − .14; *p* < .05)	
					**Blockwise regression analysis**	
					Parental attachment did not predict gambling disorder^*^	
					Peer attachment negatively predict gambling disorder (*β* = − 0.17, *T* = − 2.38, p < .05)	
Kaya and Deveci (2021)	Turkey	*n* = 110 males: 53 patients with online gambling disorder Age range = *na* (*M* = 32.08, *SD* = 7.20); 57 healthy controls. Age range = *na* (*M* = 33.51, *SD* = 6.63)	*Gambling*: SOGS; *Attachment styles*: RSQ	Trait	**Multivariate variance analysis**	16
					The patient group had significantly lower level of secure attachment (*F* = 22.720, *p* < .001) and significant higher level of dismissive (*F* = 9.484, *p* = .003) and fearful (*F* = 14.328, *p *< .001) attachment compared to the control group	
					No significant differences emerged between patient and control group on preoccupied attachment	
					**Logistic regression analysis**	
					Gambling behavior was 1.17 times higher (95% *CI*: 1.03–1.32) in presence of dismissive attachment	
					The presence of secure attachment was found to be protective (multivariate *OR*: 0.68, 95% *CI*: 0.56–0.83)	
Keough et al. (2018)	Canada	*n* = 275 participants with a lifetime diagnosis of a depressive disorder or a bipolar disorder and endorsed a mood episode within the past ten years (males = 37.1%). Age range = *na* (*M* = 43.02, *SD* = 11.58)	*Attachment*: ECR-R; *Gambling severity*: SOGS	Contextual (partner)	**Bivariate correlation**	16
					Attachment avoidance (but not anxious attachment) was positively correlated with gambling problem severity (*r* = 0.13; *p* < 0.05)	
					**Structural equation model**	
					Anxious (*B* = 0.079, 95% *CI* [0.025, 0.162]) and avoidant (*B* = 0.037, 95% *CI *[0.003, 0.116]) attachment were indirectly associated to problem gambling, via both depressive symptoms and coping motives sequentially	
Magoon and Ingersoll (2006)	United States	*n* = 116 students (Males = 43.0%)	*Attachment*:	Contextual (parents)	**Correlation**	14
		Age = 14–19 (*M* = 16.8, *SD* = 1.19)	IPPA; *Gambling Behavior*: SOGS-RA		Parent Trust and communication factor (but not alienation) was negatively associated with gambling (*r* = − 0.21, *p* < .05)	
Pace et al. (2013)	Italy	*n* = 268 male adolescents. Age = 15–17 (*M* = 16.23, *SD *= .39): 100 no gamblers [Age range = *na* (*M* = *na*, *SD* = *na*)]; 98 at-risk gamblers [Age range = *na* (M = *na*, SD = *na*)]; 70 pathological gamblers [Age range = *na* (M = *na*, SD = *na*)]	*Attachment*: RQ; *Gambling Behavior*: SOGS	Trait	**Univariate analyses of variance **	17
					At-risk and pathological gamblers reported higher level of fearful attachment compared to non-gamblers [*F*(2, 265) = 6.55, p < .001]	
					At-risk gamblers reported lower level of secure attachment compared to non-gamblers and pathological gamblers [*F*(2, 265) = 6.67, *p* < .001]	
					**Discriminant function analysis**	
					The difference between non-gamblers and at-risk gamblers was better explained by a function named “self-in-relation” (internalizing problems, fearful attachment, (lack of) secure attachment and (low) perceived support) while difference between at- risk gamblers and pathological gamblers was better explained by a function named “self-definition” (externalizing problems and preoccupied attachment)	
Ponti et al. (2021)	Italy	*n* = 80 couples: 35 clinical couples with a partner who had a severe Gambling Disorder [males = 82.86%; Age range = 31–55 (*M* = 43.91, *SD* = 6.28)]; 45 control couples [males = 73.3%; Age = 30–55 (*M* = 45.27, *SD* = 5.85)]	*Attachment*: ECR-R; *Gambling behavior*: Gambling Disorder was assessed following the DSM 5 criteria	Contextual (partner)	**Univariate analyses of variance **	16
					Pathological gamblers reported higher levels of anxious (*F*(1, 77) = 5.66, *p* = .020, *η*^*2*^ = .07) and avoidance attachment (F(1, 77) = 12.96, *p* = .001, *η*^*2*^ = .14) compared to the control group	
Terrone et al. (2021)	Italy	*n* = 178 adolescents (males = 42.1%). Age range = 16–22 (*M* = 17.51, *SD* = 0.818)	*Gambling*: SOGS-RA; *Attachment*: FFI	Trait	**Correlation analysis**	16
					Gambling was negatively associated with secure-autonomous attachment (*r* = − 0.263, *p* < 0.01) and positively associated with insecure-dismissing attachment (*r* = 0.186, *p* < 0.05)	
					No significant associations were found between gambling and insecure-preoccupied (*r* = 0.046) and disorganized-disoriented (*r* = 0.051) attachment	
					**Mediation analysis**	
					Insecure attachment was directly associated with gambling disorder (*β *= 0.669, *p *< .001). Moreover, insecure attachment negatively influenced the developmental perspective (*β* = − 0.742, p < .001), which affected the theory of mind toward one’s own best friend (*β* = 0.352, p < .001), which in turn predicted the adaptive response to distress (*β* = 0.215, p < .05), which ultimately impacted gambling (*β* = − 0.219, p < .05)	
Tonioni et al. (2014)	Italy	*n* = 80 participants: 31 Internet Addicted patients [males = 96.77%; Age range = *na* (*M* = 24.7, *SD* = 11.4)]; 11 Pathological Gamblers patients [males = 90.91%; Age = *na* (*M* = 31.6, *SD* = 9.5)]; 38 healthy volunteers [males = 94.*74%; *Age = na *(M* = 27.2, *SD* = 10.4)]	*Attachment to parents and peers*: IPPA; *Gambling behavior*: Pathological gambling was assessed following the DSM-IV criteria (APA, 2000)	Contextual (parents and peers)	**Multivariate analyses of variance**	16
					Pathological gamblers reported significantly lower levels in trust towards parents and communication with peers compared with healthy subjects (*M*(*SD*) = 28.4 + 4.4 and 32.7 + 4.5, *p* < .001; *M*(*SD*) = 24.2 + 7.7 and 32.0 + 6.6, p < .01, respectively)	

AAQ = Adolescent Attachment Questionnaire; DSM-IV-MR-J = DSM-IV-Multiple Response-Juvenile; ABQ = Addictive Behavior Questionnaire; PTI-ASS = Psychological Treatment Inventory-Attachment Styles Scale; ECR-R = Experiences in Close Relationships-Revised; KFG = Kurzfragebogen zum Glücksspielverhalten; IPPA = Inventory of Parent and Peer Attachment; SOGS-RA = South Oaks Gambling Screen-Revised for Adolescents; SOGS = South Oaks Gambling Screen; RSQ = Relationship Scales Questionnaire; RQ = Relationships Questionnaire; FFI = Friends and Family Interview; *na* = not available; * = non-significant results were not reported by the Authors

The measures used in the studies, the constructs they assessed, and the frequency of their use are listed in Table 3.

**Table 3:** Measurement instruments and dimensions listed in the included studies

Construct	Measures	Construct dimensions	Studies	No of studies
Gambling	Addictive Behavior Questionnaire—Severity Index (ABQ-SI; Caretti et al., 2016)	Unidimensional construct	Caretti et al.(2018)	1
	South Oaks Gambling Screen-Revised for Adolescents (SOGS-RA; Winters et al., 1993)	Unidimensional construct (non-problem gamblers, at-risk gamblers, and problem gamblers)	Estévez et al. (2017), Estévez et al. (2021), Magoon and Ingersoll (2006), Terrone et al. (2021)	4
	South Oaks Gambling Screen (SOGS; Lesieur & Blume, 1987)	Unidimensional construct (non-problematic gamblers, At-risk gamblers, and pathological gamblers)	Caretti et al. (2018), Kaya and Deveci (2021), Keough et al. (2018), Pace et al. (2013)	4
	DSM-IV diagnostic criteria for Pathological Gambling (APA, 2000)	Diagnosis of a Pathological Gambling if at least five of the 10 diagnostic criteria are met	Tonioni et al. (2014)	1
	DSM-5 diagnostic criteria for Gambling Disorders (APA, 2013)	Diagnosis of severe Gambling Disorder if at least 8 criteria of the 9 are met	Ponti et al. (2021)	1
	DSM-IV-Multiple Response-Juvenile (DSM-IV-MR-J; Fisher, 2000)	Unidimensional construct (non-gamblers, social gamblers, at-risk gambling, and problem gambling)	Calado et al. (2017b), Calado et al. (2020)	2
	Kurzfragebogen zum Glücksspielverhalten (KFG; Petry, 1996)	Unidimensional construct (nonpathological gambling, some gambling problem, low pathological gambling, intermediate pathological gambling, and severe pathological gambling)	Di Trani et al. (2017)	1
Attachment	Addictive Behavior Questionnaire—Seven Domains Addiction Scale (ABQ-7DAS; Caretti et al., 2016)	Separation anxiety	Caretti et al. (2018)	1
	Adolescent Attachment Questionnaire (AAQ; West et al., 1998)	Overall attachment and three dimensions: angry distress, availability, and goal-corrected partnership	Calado et al. (2017b), Calado et al. (2020)	2
	Experiences in Close Relationships-Revised questionnaire (ECR-R; Fraley et al., 2000)	Dimension: Attachment anxiety and attachment avoidance. Subgroups: Secure, Preoccupied, Dismissing, Fearful	Di Trani et al. (2017), Keough et al. (2018), Ponti et al. (2021)	3
	Friends and Family Interview (FFI; Kriss et al., 2012; Steele & Steele, 2005)	attachment classifications (secure-autonomous, insecure-dismissing, insecure-preoccupied, insecure-disorganized) and dimensional scores across numerous domains	Terrone et al. (2021)	1
	Inventory of Parent and Peer Attachment (IPPA; Armsden & Greenberg, 1987)	Overall attachment and three dimensions: trust, communication, alienation	Estévez et al. (2017), Estévez et al. (2021), Magoon and Ingersoll, (2006), Tonioni et al. (2014)	4
	Psychological Treatment Inventory—Attachment Styles Scale (PTI-ASS; Giannini et al., 2011)	Secure, Preoccupied, Avoidant, Disorganized	Caretti et al. (2018)	1
	Relationship Questionnaire (RQ; Bartholomew & Horowitz, 1991)	Secure, Dismissing, Preoccupied, Fearful	Pace et al. (2013)	1
	Relationship Scales Questionnaire (RSQ; Griffin & Bartholomew, 1994)	Secure, Dismissing, Preoccupied, Fearful	Kaya and Deveci (2021)	1

Regarding the evaluation of gambling, four studies used the South Oaks Gambling Screen-Revised for Adolescents (SOGS-RA; Winters et al., 1993), other four the South Oaks Gambling Screen (SOGS; Lesieur & Blume, 1987), two the DSM-IV-Multiple Response-Juvenile (DSM-IV-MR-J; Fisher, 2000), and one each the Addictive Behavior Questionnaire—Severity Index (ABQ-SI; Caretti et al., 2016), the DSM-IV diagnostic criteria for Pathological Gambling (APA, 2000), the DSM 5 diagnostic criteria for Gambling Disorders (APA, 2013), and the Kurzfragebogen zum Glücksspielverhalten (KFG; Petry, 1996).

Regarding attachment-related phenomena, the vast majority of studies adopted a context-specific model of attachment (*n* = 9), three studies a trait model of attachment, while one study both a context-specific and trait model. As shown, attachment related phenomena were evaluated by eight different instruments: four studies used the Inventory of Parent and Peer Attachment (IPPA; Armsden & Greenberg, 1987), three the Experiences in Close Relationships-Revised questionnaire (ECR-R; Fraley et al., 2000), two the Adolescent Attachment Questionnaire (AAQ; West et al., 1998), and one each the Addictive Behavior Questionnaire—Seven Domains Addiction Scale (ABQ-7DAS; Caretti et al., 2016), the Friends and Family Interview (FFI; Kriss et al., 2012; Steele & Steele, 2005), the Psychological Treatment Inventory—Attachment Styles Scale (PTI-ASS; Giannini et al., 2011), the Relationship Questionnaire (RQ; Bartholomew & Horowitz, 1991) and the Relationship Scales Questionnaire (RSQ; Griffin & Bartholomew, 1994).

### Main Findings

#### Adolescents and Young Adults

##### Trait Model of Attachment and Gambling

The two studies that adopted a trait model of attachment (Pace et al., 2013; Terrone et al., 2021) found a positive association between insecure attachment and gambling. In particular, Pace et al. (2013) found that at-risk and pathological gamblers reported higher level of fearful attachment style compared to non-gamblers, and at-risk gamblers also reported lower levels of secure attachment style compared to non-gamblers. Moreover, authors highlighted that the difference between non-gamblers and at-risk gamblers was better explained by a function that they called “self-in-relation”, characterized by internalizing problems, fearful attachment style, lack of secure attachment and low perceived support. Instead, the difference between at-risk gamblers and pathological gamblers was better explained by a function called “self-definition”, characterized by the presence of externalizing problems and preoccupied attachment style.

Terrone et al. (2021) found that gambling was negative correlated with secure-autonomous attachment, positively with insecure-dismissing attachment, and not correlated with insecure-preoccupied and disorganized-disoriented attachment. Furthermore, the authors found a direct effect of insecure attachment on gambling.

##### Attachment to Parents and Gambling

A total of five studies with adolescents and young adults focused on attachment to parents. In particular, three studies investigated attachment to parents by not considering them separately (Calado et al., 2017b, 2020; Magoon & Ingersoll, 2006), while two studies examined attachment to mother and father separately (Estévez et al., 2017, 2021).

Regarding the attachment to parents investigated as a whole, Calado et al. (2017b) found no statistically significant correlations between the dimensions of angry distress, availability, and goal-corrected partnership of attachment and gambling. In addition, authors found that attachment to parents did not predict gambling. Instead, Magoon and Ingersoll (2006) found that the dimension of trust and communication of attachment to parents was significantly correlated with gambling, whereas no association with the dimension of alienation was found.

Studies that disentangled attachment for mother and father have found different results. Estévez et al. (2017) found that gambling disorder was negatively correlated to attachment to father but not to mother, while Estévez et al. (2021) found a negative correlation between gambling and attachment to both parents. In addition, Estévez et al. (2021) reported that problem gamblers showed lower levels of attachment to father and mother than non-problem gamblers. However, similarly to Calado et al. (2017b), Estévez et al. (2017) found that neither father nor mother attachment predicted gambling disorder.

##### Attachment to Peers and Gambling

Only two studies investigated the relationship between attachment to peers and gambling (Estévez et al., 2017, 2021). Estévez et al. (2017) found that lower levels of peer attachment predicted gambling disorder. However, in their subsequent study, Estévez et al. (2021) found no significant differences in the levels of peer attachment between problem gamblers and non-problem gamblers.

#### Adults

##### Trait Model of Attachment and Gambling

The two studies that investigated the relationships between trait model of attachment and gambling found conflicting results. In particular, Caretti et al. (2018) did not find a significant correlation between separation anxiety, reflecting an insecure attachment, and gambling. Differently, Kaya and Deveci (2021) found that patients with online gambling disorder reported significantly lower level of secure attachment style and significant higher level of dismissive and fearful attachment styles compared to the control group, whereas no statistically significant differences between the groups emerged regarding preoccupied attachment style. Furthermore, the logistic regression analysis showed that the gambling behavior was 1.17 times higher in the presence of a dismissive attachment style, while a secure attachment style was found to be a protective factor.

##### Attachment to Parents and Gambling

Only one study examined attachment to parents (Tonioni et al., 2014), without considering the distinction between attachment to mother and attachment to father, and found that pathological gamblers reported significantly lower levels of trust towards parents than healthy subjects.

##### Attachment to Peers and Gambling

Attachment to peers was taken into consideration only in one study that highlighted significant differences between pathological gamblers and healthy subjects (Tonioni et al., 2014). Specifically, pathological gamblers reported significantly lower levels in communication with peers compared to healthy subjects.

##### Attachment to Partner and Gambling

Attachment to partner was considered by four studies and an overall association was found between partner attachment and gambling, with one exception. In particular, only one study found that the secure, preoccupied, avoidant and disorganized attachment styles did not correlate with gambling (Caretti et al., 2018). In contrast, the other study found that gamblers presenting with a fearful attachment style reported greater gambling severity than those with a secure one (Di Trani et al., 2017).

Regarding the two higher-order dimensions of attachment, attachment avoidance and attachment anxiety were found to be positively correlated with gambling in all studies (Di Trani et al., 2013; Di Trani et al., 2017; Ponti et al., 2021), with the exception of the Keough et al. (2018)’s which did not find associations with attachment anxiety.

#### Studies that Considered the Possible Mediators of the Relationships Between Attachment and Gambling

Five studies explored the presence of possible mediators of the relationship between attachment and gambling. In general, studies showed that the relationship between attachment and gambling appears to be mediated by different mediators depending on the attachment model (trait or context-specific model) and the specific relationship taken into consideration (e.g., parents or partner).

One study took into consideration the trait model of attachment in adolescents and young adults (Terrone et al., 2021). Such a study highlighted a direct and indirect effect of insecure attachment on gambling through a chained mediation: insecure attachment negatively affects the developmental perspective, which in turn influences the theory of mind toward one's own best friend, this affects the adaptive distress response, and ultimately impacts gambling.

Three studies have included possible mediators considering the context-specific model of attachment. Two studies looked at parents as a whole and found that the attachment dimension of angry distress was indirectly associated with problem gambling through emotion-focused coping (Calado et al., 2017b). Moreover, attachment dimensions of angry distress, availability, and goal-corrected partnership were indirectly associated with problem gambling through sensation-seeking in both the Portuguese and the English samples (Calado et al., 2020). The third study looked at attachment to mother and father separately and highlighted that one aspect of alexithymia – the difficulty in identifying emotions – fully mediates the association between attachment to mother and gambling (Estévez et al., 2021).

One study considered the context-specific model of attachment with reference to partner in adults and highlighted that both anxious and avoidant attachment were indirectly associated to problem gambling through a chained mediation: insecure attachment positively influences depressive symptoms which, in turn, affect coping motives for gambling, which ultimately impact the severity of gambling (Keough et al., 2018).

## Discussion

Although attachment appears to play a significant role in the etiopathogenesis of addictive behaviors, (D'Arienzo et al., 2019; Musetti et al., 2022; Schindler & Bröning, 2015; Schindler, 2019), to our knowledge no previous systematic literature review has addressed this important issue with regards to gambling. Therefore, the present study fills this gap in current knowledge by offering a systematic review of the available literature on the relationships between attachment and gambling, also reporting the mediators of this relationship highlighted by the literature. Although the vast majority of the included studies neither adequately justify the sample size (e.g., with power analysis) nor address and classified the non-responders, the quality assessment highlights an overall low risk of bias of the studies included and therefore the results are suitable to provide evidence-based conclusions. Indeed, all the studies included in this systematic review showed high quality, with the exception of one study (Magoon & Ingersoll, 2006) which nevertheless reported medium quality.

Despite some exceptions, the present systematic review underscores that attachment plays a role in gambling behaviors. The most important results are related to the fact that while secure attachment represents a protective factor for the onset of gambling behaviors, insecure attachment turns out to be a vulnerability factor in dual form. In fact, on the one hand, insecure attachment directly favors gambling behaviors; on the other, it influences certain psychological characteristics that in turn predict gambling behaviors. Below we will discuss the results in more detail, taking into consideration the model used to define attachment (i.e., trait versus context-specific model) and population age.

### Findings Based on the Trait Model of Attachment

Overall, studies which used the trait model of attachment report a negative association between secure attachment and gambling as well a positive association between insecure attachment and gambling in both adolescents/young adults and adults. The only exception is the study conducted by Caretti et al. (2018) which failed to find a correlation between attachment and gambling. Furthermore, concordant results were found regarding the specific role played by the different types of insecure attachment, with few exceptions. Consistent results were reported regarding the positive association between dismissive attachment and gambling, so much so that gambling behavior results to be 1.17 times higher in the presence of this type of attachment (Kaya & Deveci, 2021). This finding is in line with the literature which pointed out that dismissive attachment appears to be a significant risk factor for externalizing problems, since individuals with dismissive attachment defensively divert attention from their emotional distress by coping with it through acting out (De Santis et al., 2021; Ramos et al., 2016). Conversely, overall preoccupied attachment was not found to be associated with gambling (e.g., Kaya & Deveci, 2021; Terrone et al., 2021) with few exceptions. Among adolescents and young adults, Pace et al. (2013) found that the difference between non-gamblers and at-risk gamblers was explained by the lack of secure attachment in the latter, while the difference between pathological and at-risk gamblers was explained by higher levels of preoccupied attachment in the former. Therefore, it is possible to suppose that secure attachment is a protective factor for the implementation of gambling behaviors per se, on the one hand. On the other hand, it is the specific characteristics of a preoccupied attachment (such as ambivalence towards closeness, the craving for attention from others and the need for recognition and entitlement) that can lead to problematic gambling behavior in adolescence. Finally, less strong evidence was found for fearful/disorganized attachment, with some studies showing a positive association with gambling (Kaya & Deveci 2021; Pace et al., 2013) and some others that did not find no association (Terrone et al., 2021). Considering the foregoing, future studies might want to examine the motivations for gambling and the preferred type of gambling activity to better investigate the relationship between the different types of attachment (especially fearful/disorganized) and such behaviors. Indeed, it is possible to suppose that based on the specific characteristics of the different types of insecure attachment, individuals can resort to gambling in order to satisfy their specific attachment needs and choose certain types of games rather than others.

Furthermore, when the role played by possible factors that can intervene in this relationship is taken into consideration, the results highlighted both direct and indirect effects of insecure attachment on gambling behaviors (Terrone et al., 2021). Specifically, Terrone et al. (2021) highlighted that insecure attachment negatively influences some central aspects in the adolescent's adaptation, such as the development perspective, theory of mind toward one's best friend and adaptive response to stress, which in turn are linked to each other by a sequential influence.

### Findings Based on the Attachment to Parents

Considering that parents are among the main reference figures during youth, it is not surprising that the studies that examined the relationship between attachment to parents and gambling were conducted exclusively on adolescents and young adults, with just one exception (Tonioni et al., 2014). Overall, results revealed that there is no consistent association between insecure attachment to parents and gambling. In fact, although negative correlations were found between gambling and some positive aspects of attachment, such as a good quality of communication and trust towards parents (e.g., Magoon & Ingersoll, 2006; Tonioni et al., 2014), the studies reviewed did not find a direct predictive role of attachment to parents, either as a whole or considering separately mother and father, on gambling behaviors.

However, when the presence of possible mediating variables was taken into consideration, the results consistently show an indirect effect of insecure attachment to parents on gambling behaviors via coping focused on emotions (Calado et al., 2017b), sensation-seeking (Calado et al., 2020), and difficulty in identifying emotions (Estévez et al., 2021). Therefore, it is possible to assume that an insecure attachment to parents may adversely affect the development of an adaptive psychological functioning, such as a tendency to search for strong emotions and a poor ability to identify and manage affective states, which in turn leads to a greater likelihood of resorting to maladaptive behavior such as gambling.

### Findings Based on the Attachment to Peers

Although it is widely demonstrated in the literature that the relationship with peers has a central role in the life of adolescents and young adults (Fraley & Davis, 1997; Majorano et al., 2015), surprisingly only two studies have investigated this aspect in relation to gambling behavior in this population, while only one study looked at adults (Tonioni et al., 2014). Conflicting results were reported (Estévez et al., 2017, 2021), and this might be due to the fact that specific attachment subdimensions (i.e., alienation, trust, and communication) were not taken into consideration and disentangled. Results coming from systematic reviews on attachment and other behavioral addictions have shown that especially alienation from peers is associated with behavioral addictions tendencies among young people (Musetti et al., 2022). In keeping with these findings, it might be the case that it is the presence of feelings of isolation, anger and detachment experienced in the relationships with peer that favor the onset or maintenance of gambling behavior since adolescent problem gamblers report poor social support from peers and a sense of unpopularity within the class group (Delfabbro et al., 2006; Hardoon et al., 2004). Future studies on adolescents and young adults could focus their attentions on the link between attachment subdimensions and gambling behaviors in order to clarify this issue.

### Findings Based on the Attachment to Partner

Attachment to the partner appears to be the type of relationship most explored in the adult population, and this is not surprising since partner is the main reference figure in adulthood. In general, all the studies showed an overall positive association between insecure attachment to partner and gambling (Di Trani et al., 2017; Keough et al., 2018; Ponti et al., 2021), with only one exception (Carretti et al., 2018).

Regarding attachment styles, individuals with a fearful attachment style report greater severity of gambling behavior than those with a secure one (Di Trani et al., 2017). Individuals with fearful attachment are characterized by intense distrust of others and by a search for closeness with others but at the same time by an avoidance due to the fear of rejection and the vision of oneself as unlovable and unworthy of care. These findings are generally explained by claiming that for individuals with fearful attachment gambling is a way for regulating their constant state of internal disorganization. However, gambling at the same time strengthens this state as in the event of a win the individual perceives a momentary false sense of power and control, but when there is a loss the belief of inadequacy is confirmed.

Considering the two higher-order dimensions of attachment, namely anxiety and avoidance, these indices of insecurity have been positively correlated with gambling in most studies. Since individuals with high levels of attachment anxiety show low self-control and an inability to regulate their emotional states by overreacting to them (Mikulincer et al., 2003; Tangeny et al., 2004), it has been suggested that such individuals engage in gambling as a way of coping with their own discomfort (Di Trani et al., 2013). Moreover, both anxiety and avoidance were indirectly associated with problem gambling through a chained mediation: insecure attachment predicts greater depressive symptoms, which in turn affects coping motives for gambling, which ultimately leading to greater gambling severity (Keough et al., 2018).

### Limitations

Although this systematic review provides an important contribution to the understanding of the relationship between attachment and gambling, it also has limitations. Firstly, we only included studies published in English and this did not allow for any significant contributions published in other languages ​​to be considered. Secondly, all the included studies have been conducted in Western countries and this may hinder the generalizability of the results since culture plays a non-negligible role in the formation of attachment bonds (Van Ijzendoorn & Kroonenberg, 1988). Third, the present review is based exclusively on cross-sectional studies and therefore it was not possible to establish the direction of the association between attachment and gambling, especially in reference to the context-specific model of attachment. A further limitation is that the studies included mostly used self-reported measures that are susceptible to response bias. Moreover, the included studies involved convenience samples, and this could affect the generalizability of the results as these types of samples might not be representative of the general population. A further limitation is that we only considered studies that used a conceptualization of attachment strictly consistent with Bowlby's theory. Therefore, constructs closely related to attachment (i.e., bonding) were not taken into consideration. "Attachment" and "bonding" are often used interchangeably in the literature and this confusion stems from the fact that they both describe different aspects of the same phenomena, namely how relationships are formed and how they affect the development of the child (Ettenberger et al., 2021). However, among the various points of divergence of these two concepts, one of the main ones is on whom the focus is laid: while attachment refers to how the child builds the relationship with the caregivers, bonding describes the parent's feelings, thoughts, and behaviors towards the child (Ettenberger et al., 2021). Therefore, it would be interesting and useful if future systematic reviews took into consideration the role of bonding in gambling in order to have a broader picture of how the relationship with significant others influences the implementation of maladaptive behavior from different points of view.

Conflicting results might be at least part explained by the different focus of the studies. Indeed, the reviewed studies relied on different measures of attachment and gambling, and in some studies specific attachment sub-dimensions were not disentangled. Moreover, controversial results might be consequence of the heterogeneity of the gamblers population. In fact – and this is particularly relevant for the generalizability of our results to the gamblers population – it is well known that gambling is multifactorial and thus no single pathway can fully explain its origins (Blaszczynski & Nower, 2002): it is plausible that the pathway linking attachment to gambling concerns the so-called *emotionally vulnerable gamblers* (i.e., gamblers displaying premorbid psychopathology including anxiety, depression, insufficient problem-solving skills, and negative family experiences), whilst it might be less relevant for explaining other subtypes of gambling (e.g., *behaviorally conditioned gamblers*, see Blaszczynski & Nower, 2002). However, the reviewed studies did not identify gambler subtypes. This might explain conflicting results concerning the role of insecure attachment among adults in that we cannot rule out that some samples mainly consisted of emotionally vulnerable gamblers, whilst some others largely included behaviorally conditioned gamblers.

### Implications

Some implications for future research can be provided on the basis of the aforementioned limitations. Specifically, since it is plausible to assume that engaging in gambling behaviors reinforces and exacerbates the negative perception that an individual has of their attachments towards significant others (parents, friends and/or partners), who at first contributed to the onset of such behaviors, it is important to conduct longitudinal studies in this field to better understand this relation. Moreover, future studies should investigate both attachment and gambling through clinical interviews, even considering that some instruments used a past-year timeframe, whereas others used a lifetime perspective (which produces higher problem gambling prevalence rates). Finally, further studies that simultaneously take into consideration the different attachment figures (mother, father, peers, and partner) are essential to better understand the distinct role played in the onset and maintenance of gambling behaviors, also according to the life phase (e.g., adolescence, young adulthood, adulthood, old age) and culture (western and eastern).

Findings suggested that attachment might be a factor associated with gambling in two ways. On the one hand, especially young individuals with insecure attachment appear to be more prone to engage in gambling behaviors; on the other hand, insecure attachment indirectly affects gambling behaviors by affecting a range of individual characteristics which in turn favor such behaviors. For example, insecure attachment fosters a tendency to pursue new and different sensations, feelings and experiences and hinders an individual's ability to identify their feelings. Consequently, individuals with insecure attachment may engage in gambling behaviors driven by curiosity for new experiences and a desire for excitement resulting from the unpredictability of these experiences or as a way of coping with their own feelings that they are unable to define clearly. As insecure attachment is resistant to change (Fletcher et al., 2015) clinicians might be advised to focus their attention on more easily modifiable variables (i.e., the mediators) that can be targeted in a perspective of prevention and treatment of gambling. From a preventive perspective, interventions might be directed to those at risk of developing a gambling problem and/or to the wider relationship network. With those at risk, interventions could focus both on the individual psychological characteristics that characterize gamblers and on promoting the relational well-being of individuals, fostering positive relationships with significant others, such as encouraging the expression and sharing of one's own unease. With a wider relationship network, the interventions could be aimed at promoting parenthood in order to foster a relationship characterized by understanding, responsiveness and emotional reactivity in order to prevent the onset of gambling problems among adolescents.

## Data Availability

Data sharing is not applicable to this article as no datasets were generated or analyzed during the current study.
